# Dermatophytosis and its risk factors among children visiting dermatology clinic in Hawassa Sidama, Ethiopia

**DOI:** 10.1038/s41598-023-35837-7

**Published:** 2023-05-27

**Authors:** Mengistu Haro, Tsegaye Alemayehu, Abraham Mikiru

**Affiliations:** 1 Department of Biology, Hawassa University College of Computational Sciences, Hawassa, Sidama Ethiopia; 2 School of Medical Laboratory Science, Hawassa University College of Medicine and Health Sciences, P.O. Box: 1560, Hawassa, Sidama Ethiopia

**Keywords:** Risk factors, Fungi, Infectious-disease diagnostics

## Abstract

Dermatophytosis represents one of the common fungal diseases that attack the skin, hair and nail of human beings worldwide. It causes chronic morbidity in children and the condition is more common, in developing countries. The study aimed to determine dermatophytosis and its associated factors among children in Hawassa Sidama, Ethiopia April 2021–October 2021. A cross-sectional study was conducted on children suspected of cutaneous fungal infections. Data were surveyed based on a semi-structured questionnaire. Standard laboratory methods were used to identify the dermatophytes. The data entry and analysis were conducted with SPSS version 26. The Chi-square test was used to check the predictor and a p-value < 0.05 was taken as a significant value. A total of 83 study subjects included in the study in which all 83 (100%) patients were positive for fungal elements (hyphae/and spores) in microscopy, of this 81 (97.6%) yielded growth on culture. Hair scalps 75 (90.4%) were the dominant among the case. *Trichophyton* 52 (62.6%) was the dominant aetiology followed by *Microsporum* 22 (26.6%). Intervention measures to tackle dermatophytosis should emphasis on tinea capitis among 6–10 years old children with history of recent migration by raising awareness of communities through health extension programs.

## Introduction

Superficial fungal skin infections, dermatophytosis, is one of the most common fungal diseases globally that lead to chronic morbidity, particularly in developing nations^1^. Dermatophytosis has a specific designation depending on the anatomical site of infection, such as *Tinea capitis* (on the head) *Tinea corporis*, (on the trunk or non-hairy body parts) or *Tinea pedis* (on feet or athletes’ foot). *Tinea capitis* is one of the most common public health concern among children in developing nations^2^. It is common in children with incidence increasing in summer (the rainy season between May and August) and declining in winter^3^ (dry season).

High temperature and humidity are the factors that are favourable to increased incidence^4^. The topographical setting, health care, immigration, climate (temperature, humidity and wind), overcrowding, environmental sanitation culture, age of individuals, personnel hygiene and socioeconomic situations have been described as the main issues for the spread of the disease^5,6^. The climatic circumstances and seasonal disparities have a direct influence on the incidence of certain dermatophytosis infections^7^.

Dermatophytosis infections are more common in tropical and sub-tropical regions because of high temperatures and humidity^7^. Apart from climatic conditions, the congregation of a large number of people and their poor standards of hygiene facilitates the transmission among children between the ages of 4 and 16 years. This is because of the increased contact with different sources and having an inadequate amount anti fungal fatty acids synthesized in their sebum that predispose them to infections^3,8,9^.

The most common etiologic agents of dermatophytosis are *Trichophyton*, *Microsporum* and *Epidermophyton* and infection may also be caused rarely by the members of the *genus Candida* and by non-dermatophytic moulds belonging to the genera *Fusarium*, *Scopulariopsis* and *Aspergillus*^3^. *Trichophyton, Microsporum* and Epidermophyton are (worldwide) in geographical distribution^10^. According to the World Health Organization survey on the incidence of dermatophytes infection, about 20% of the people worldwide present with cutaneous infection from the genus the dominant species are *Tinea corporis* or *Tinea circinate* followed by *Tinea cruris* and *Tineapedis*. *Tinea corporis* accounts for about 70% of dermatophytic infections^11^.

Ethiopia is a developing nation located in the tropical region where it experiences a high prevalence of dermatophytosis. The country is known for a diversity of ecoclimatic zones, wet lands, rivers, chains of large lakes situated in the great east African rift valley. People residing in the shores of big lakes like Hawassa experience high temperatures coupled with high humidity that are known to be conductive for the transmission of dermatophytosis^9^. As far as our knowledge is concerned, published data are scarce on the etiological agents and associated factors of dermatophytosis in the study area.

## Material and methods

### Study design, area, and period

A Hospital-based cross-sectional study was conducted at Hawassa University Comprehensive Specialized Hospital (HUCSH) and Dr Girum's Dermatology clinic in Hawassa city, Sidama Regional State, Ethiopia from April 2021 to October 2021.The hospital was has more than 300 beds and gives service to 10–12 million people. Hawassa city is located in southern Ethiopia, on the shores of Lake Hawassa which is one of the Great Rift Valley lakes and is located 273 km south of Addis Ababa the city lies on latitude and longitude of 7°3′N–38°28′E, has an elevation of 1708 m above sea level. The climate is characterized by annual average rainfall of 945 mm, average temperature of 19.5 °C and a humidity of 70–80%^12^. The University Hospital is the only biggest comprehensive specialized and teaching hospital in the region and consists of an operating room, intensive care unit (ICU), 16 wards and 11outpatient departments the study was carried out at a dermatology clinic and laboratory work in the medical microbiology laboratory of health science of college of medicine and health science. Dr Girum Dermatology Clinic is a medium clinic which is found on dermatological cases in Hawassa City.

### Population of the study

All children who visited the two institutions during the study period were the source population and children clinically suspected or have signs and symptoms of dermatophytosis were study subjects.

### Sampling techniques

A convenience sampling technique was used, by the inclusion of all volunteers who were clinically suspected cases of dermatophytosis and diagnosed as such by physical examination of the attending physicians. Accordingly, consecutive 83 children suspected of dermatophytosis were included in the study.

### Study variables

Culture-confirmed dermatophytosis is the dependent variable for this study and the independent variable includes the age, sex of the child, geographical location, site infected, personal health culture, socio-economic status, personnel hygiene, the population in place, lack of clean water, migration, family size, living and playing with pets.

### Eligibility criteria

Children clinically suspected of dermatophytosis, who were attending the two facilities during the study period and whose parents consented to participate in the study were included. Children whose parents were not willing to participate, and who had received oral or topical antifungal treatment within the previous two weeks were excluded from the study.

### Data collection procedures

During the visit to the dermatology clinic, the attending clinician/nurse along with the investigator explained the objective of the study to each patient or parent/caretaker and written formal consent was secured. After that, the questionnaire was administered through face-to-face interview with the caregiver.

### Collection of dermatological specimens

**Figure 1:**
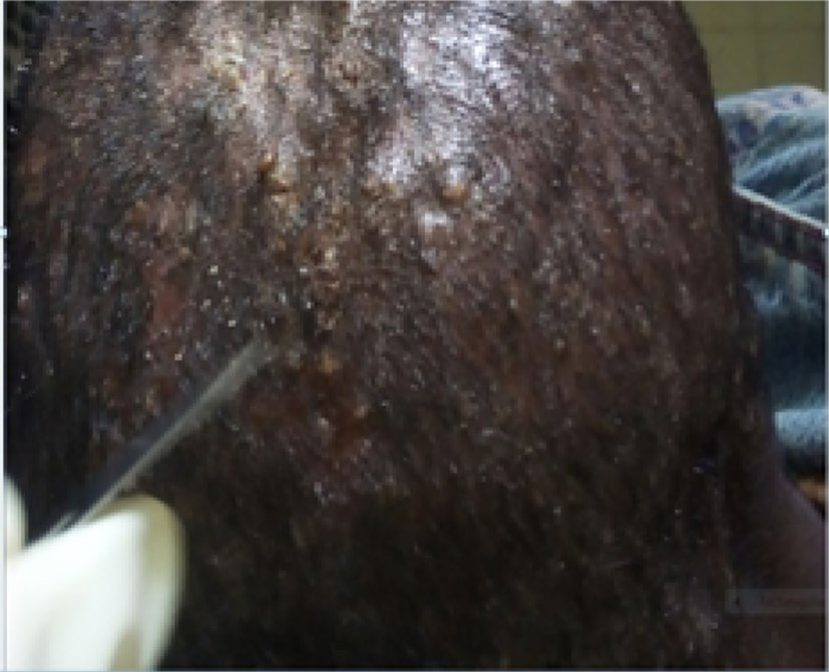
*Tinea capitis* on 3 years child.

**Figure 2:**
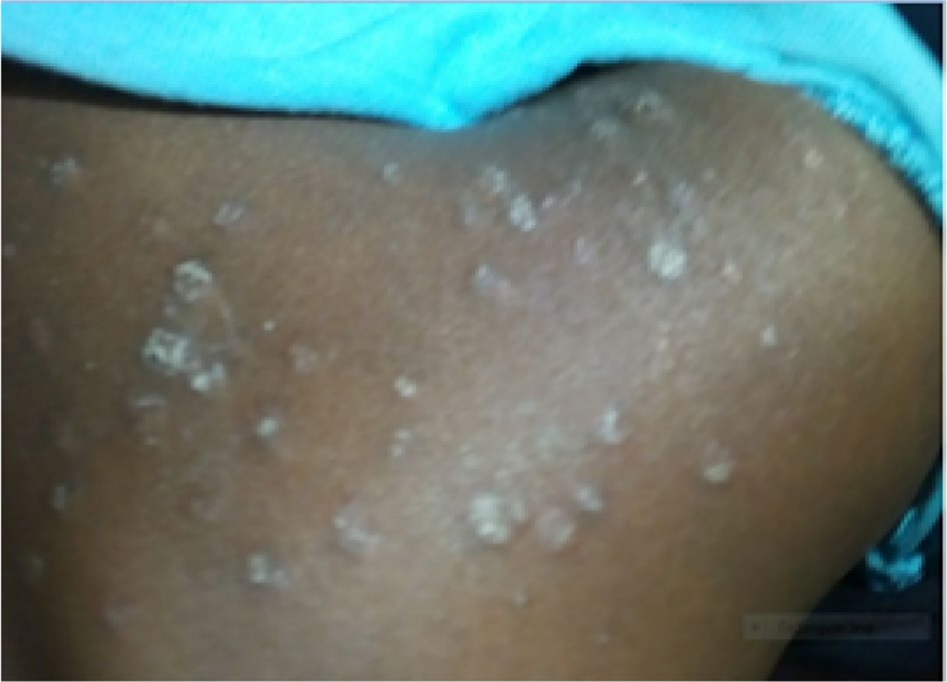
*Tinea corporis* on 7 years male child.

**Figure 3:**
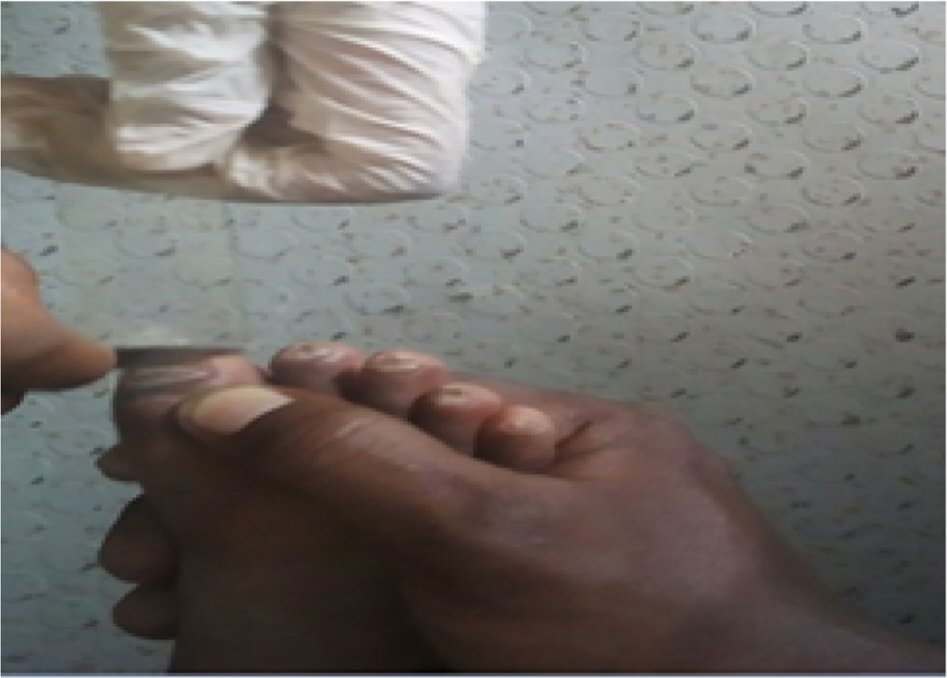
*Tinea unguium* from 6 years female child.

The dermatological sample collections were based on a standard practical guide and atlas for the diagnosis of fungal infections^13^. Briefly, after cleaning the area of the lesion on the skin with 70% ethanol, samples were taken from the erythematous, peripheral and actively growing margins of the skin lesions hair and nail by scraping with a blunt scalpel blade by trained laboratory technologists and clinical nurses. The specimens were then, transferred into a sterile Petri dish, and labelled with the patient’s code, age, sex, date of sample collection and site of infection. The samples were transported to the Microbiology Laboratory of the School of Medical Laboratory Science, College of Health Sciences within an hour of collection (Figs. 1, 2 & 3).

### Microbiological laboratory analysis

Microbiological laboratory analysis was based on the study Nagar, S.N., *Diagnosis of dermatophytosis*^14^. Each sample was treated with 1–2 drops of 10% KOH solution for 15–30 min and microscopically examined for the presence of fungal elements (spore and hyphae) under a light microscope at 10 × and 40 × magnification power. Portions of the specimens were aseptically inoculated on plates of Sabouraud’s Dextrose agar (SDA)and incubated at room temperature (22–25°°C). The SDA platecultures were periodically examined for growth of dermatophytes every other day for four weeks and positive cultures were examined macroscopically for species identification based on Colony characteristics (macroscopic)—Gross colony features observed on SDA include the colour of the surface, the colour of reverse, the texture of the surface (powdery, granular, velvety, or fluffy) type of folding (radial, cerebriform), and the rate of growth^13^.

### Data management and quality assurance

Media were checked for growth of non-dermatophytes by incubating at 25 °C for four weeks. Phenotypic identification of fungal isolates was done by a panel of experienced laboratory technologists (three) and a colour atlas of medical mycology.

### Data processing and analysis

All data were coded and entered into logbooks and then into the computer and were analyzed using IBM SPSS Statistics for Windows Version 26.0 (IBM Corp., Armonk, NY, USA). In the descriptive study, categorical variables were represented as the mean ± standard deviation (SD), and qualitative variables were expressed as relative frequencies and percent. The Chi-square test was used to compare the prevalence among the independent variables. A *p*-value of < 0.05 was considered tohave statistical significance.

### Ethical consideration

Ethical clearance was obtained from the Institutional Review Board (IRB) Department of Medical Laboratory Science, College of Health Sciences Hawassa University. Permission was also obtained from Hawassa university comprehensive specialized Hospital and Dr Girum Medium Clinic. Parents or guardians signed a consent form after being informed of the objectives of the study and the confidentiality of participants’ personal information was protected as rights to refuse to take part in the study as well as to withdraw at any time during the study period were given. All the information obtained from the study patients was coded to maintain confidentially. When the participants were found positive for dermatophytosis they were reported to the hospital and the clinician treated them accordingly. All methods were performed following the relevant guidelines and regulations.

## Result

### Sociodemographic characteristics of study participants

In this study, a total of the 83 dermatophytes suspected patients were involved consisting of 56 (40 Females and 16 Males) from a private dermatology clinic and 27 (11 Females and 16 Males) from the HUCSH dermatology clinic. Of these, 51 (61.4%) were females and 32 (38.6%) were males. The majority of the participants 45 (54.2%) were in the age range of 6–10 years followed by the age range of 1–5 years (34.9%) and 11–15 years (10.8%).Concerning residence, 69 (83.1%) were from urban and 14 (16.9%) from rural locations and in terms of occupation, 39 (47.0%) of the mothers were government employees, 35 (42%) were merchants and 9 (10.8%) were farmers (Table 1). According to educational status, 38 (45.8%) of the mothers were secondary level, 36 (43.8%) higher level and the remaining 9 (10.8%) were elementary educational level. The majority of patients 43 (51.8%) took baths only once a week, 34 (41%) twice a week and 6 (7.2%) thrice a week. Likewise, the personal hygiene of mothers/guardians was also assessed based on this 25.3% of them bathed once a week, 37.3% bathed twice a week, 30.1% of them thrice a week and 7.2% four times a week. With regards to water usage, 70 (84.3%) the participants used pipe water, 11 (13.3%) well water and the rest 2 (2.4%) used other sources (Table 1).

**Table 1:** Shows the frequency, rate of growth and associated factors among children visiting a dermatology clinic in Hawassa Sidama Ethiopia.

Variables	Frequency (%)	Dermatophytes		χ^2^, df, p-value
		Growth (%)	No growth (%)	
Sex				
Female	51 (61.4)	50 (61.7)	1 (50)	0.11, 1, 0.74
Male	32 (38.6)	31 (38.3)	1 (50)	
Age (years)				
1–5	29 (34.5)	29 (35.8)	0 (0)	16.85, 2, 0.00*
6–10	45 (54.2)	45 (55.6)	0 (0)	
11–15	9 (10.8)	7 (8.6)	2 (100)	
Residence				
Rural	14 (16.9)	14 (17.3)	0 (0)	0.42, 1, 0.52
Urban	69 (83.1)	67 (87.7)	2 (100)	
Mothers educational				
Elementary	9 (10.8)	9 (11.1)	0 (0)	0.25, 2, 0.88
Secondary	38 (45.8)	37 (45.7)	1 (50)	
Tertiary	36 (43.4)	35 (43.2)	1 (50)	
Mothers occupation				
Gov. employer	39 (47)	38 (46.9)	1 (50)	0.26, 2, 0.88
Farmer	9 (10.8)	9 (11.1)	0 (0)	
Merchant	35 (42.2)	34 (42.0)	1 (50)	
Frequency of bath for children				
Three times per week	6 (7.2)	6 (7.2)	0 (0)	0.19, 2, 0.91
Two times per week	34 (41)	33 (40.7)	1 (50)	
Once per week	43 (51.8)	43 (51.8)	1 (50)	
Source of water				
Pipe water	70 (84.3)	68 (84)	2 (100)	0.38, 2, 0.83
Well water	11 (13.3)	11 (13.6)	0 (0)	
Other	2 (2.4)	2 (2.5)	0 (0)	
Frequency of bath for guardians				
4 times per week	6 (7.2)	5 (6.2)	1 (50)	6.74, 3, 0.08
3 times per week	25 (30.1)	24 (29.6)	1 (50)	
2 times per week	31 (37.3)	31 (38.3)	0 (0)	
Once per week	21 (25.3)	21 (25.9)	0 (0)	
Family size (in person)				
1–3	26 (31.3)	26 (32.1)	0 (0)	0.94, 1, 0.33
4–6	57 (68.7)	55 (67.9)	2 (100)	
Site of infections				
Hair	75 (90.4)	74 (91.4)	1 (50)	9.14, 2, 0.01*
Skin	4 (4.8)	3 (3.7)	1 (50)	
Nail	4 (4.8)	4 (4.9)	0 (0)	
The season of sign & symptom				
Rainy/kiremt	44 (53)	42 (51.9)	2 (100)	1.82, 1, 0.40
Humid season	33 (39.8)	33 (40.7)	0 (0)	
Other	6 (7.2)	6 (7.4)	0 (0)	
Used anti-fungal cream				
No	14 (16.9)	14 (17.3)	0 (0)	0.42, 1, 0.52
Yes	69 (83.1)	67 (82.7)	2 (100)	
Migrant				
No	21 (25.3)	21 (23.5)	2 (100)	6.051, 1, 0.01*
Yes	62 (74.7)	62 (74)	0 (0)	
The same case at home				
No	73 (88)	71 (87.7)	2 (100)	0.28, 1, 0.60
Yes	10 (12)	10 (12.3)	0 (0)	
Presence of dogs & cats				
No	11 (13.3)	11 (13.6)	0 (0)	0.31, 1, 0.58
Yes	72 (86.7)	70 (86.4)	2 (100)	
Sharing a bathroom?				
No	4 (4.8)	4 (4.9)	0 (0)	0.104, 1, 0.74
Yes	79 (95.2)	77 (95.1)	2 (100)	
Walk on barefoot				
No	58 (69.9)	57 (70.4)	1 (50)	0.39, 1, 0.54
Yes	25 (30.1)	24 (29.6)	1 (50)	

*p ≤ 0.05.

### Behavioural and environmental factors among the study patients

The majority (68.7%) of the study participants were within the family size of 4–6. The peak season of the onset of dermatophyte infection among the study participants was kiremt (Major rainy season, June to September) 44 (53%) followed by Belg (the hot humid/second rainy season, April and May) 33 (39.8%) and Bega/winter (October) season 6 (7.2%) (Table 1). In most of the study participant’s home 73 (88%), there were no similar cases with patients. On the other hand, 62 (74.7%) said they came from another place (migrant), and 72 (86.7%) kept dogs and cats. The majority of patients shared bathrooms 79 (95.2%) and 58(69.9%) said they did not walk on barefoot (Table 1).

### The dermatophytosis and site of infection

Of the total of 83 study participants dermatological specimens 75 (90.36%) were hair/scalp, and 4 (4.82%) each were skin and nail samples. All of the 83 (100%) samples were positive with KOH wet mount showing fungal hyphal elements/and spores under a light microscope. And 81 (97.59%) of the specimens showed colonial growth of dermatophytes on SDA in between 1 and 4 weeks of incubation.

### The etiological agents of dermatophytosis

**Figure 4:**
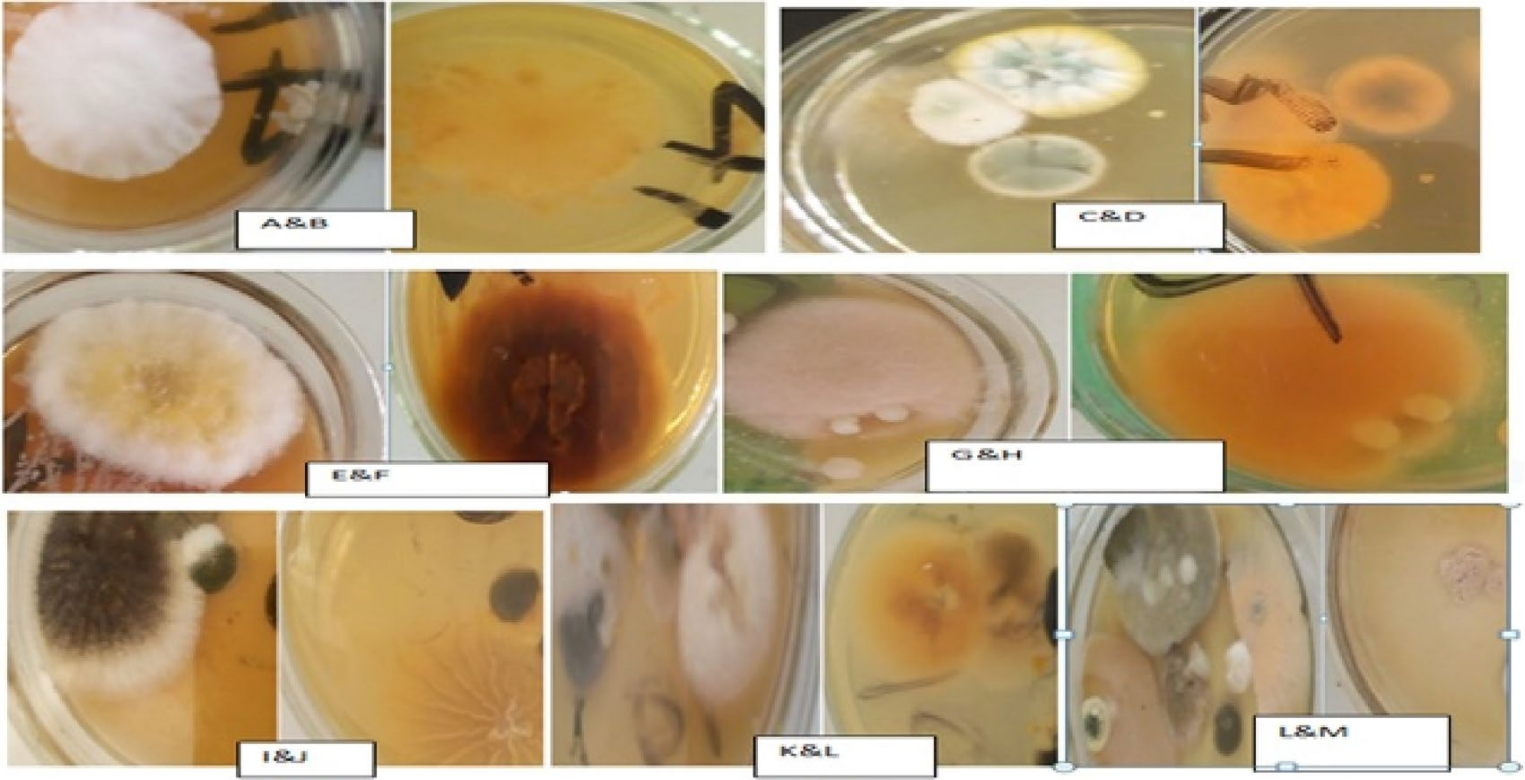
Photographs displaying the front and reverse view of gross colonial morphology of dermatophytes isolated from dermatological specimens among children in Hawassa. (**A**, **B**) Epidermophyton, (**C**, **D**) Microsporum, (**E** & **F**, **G** & **H**, **I** & **J**, **K** & **L** and **L** & **M**) Trichophyton in ascending order of their dominance.

Based on microscopic KOH wet mount and colonial morphology of culture the etiological agents for 81 of the cases were putatively identified into three genera of dermatophytes. Accordingly, the dominant genera were *Trichophyton* 52/83 (62.6%), *Microsporum* 22/83 (26.5%) and *Epidermophyton* 7/83 (8.5%). Two of the specimens did not yield growth on SDA despite the observed fungal hyphal elements on KOH microscopic mount (Table 2, Fig. 4).

**Table 2:** Distribution of dermatophytes by site of infection.

Specimens	Dermatophyte				Total
	*Epidermophyton* (%)	*Microsporum* (%)	*Trichophyton* (%)	No growth (%)	
Hair	5 (6.7)	21 (28)	48 (64)	1 (1.3)	75 (100)
Skin	2 (50)	1 (25)	0 (0)	1 (25)	4 (100)
Nail	0 (0)	0 (0)	4 (100)	0 (0)	4 (100)
Total	7 (8.4)	22 (26.6)	52 (62.6)	2 (2.4)	83 (97.6)

### Distribution of the dermatophyte species by anatomical site of infection

The distribution of the dermatophyte species by anatomical site of infection is summarized in table two (Table 2). Of the total 83 samples, 75 (90.36%) were isolated from hair specimens, and four (4.82%) each were from skin and nail samples. The most frequent genera of dermatophytes encountered in hair samples were *Trichophyton* 48/75 (64%), followed by *Microsporum* 21/75 (28%) and *Epidermophyton* 5/75 (6.6*%)*. With regard to isolates from skin *Epidermophyton 2/4 and Microsporum ¼ (25%) prevailed while all the isolates from nail belonged to Trichophyton* 4/4 (100%).

### Distribution of the dermatophytes by age and sex of the study subjects

The majority of the dermatophytes occurred among the age group of 6–10 years old 45/83 (54.22%) followed by those of 1–5 years old 29/83 (34.94%) and 11–15 years old 7/83 (8.43%). The most frequent dermatophytes encountered among the age group of 6–10 years old patients were *Trichophyton 29/45 (64.4%), Microsporum 12/45 (26.6%) and Epidermophyton 4/45 (8.8%).* On the other hand, the most frequent dermatophytes encountered among the age group of 1–5 years old patients were *Epidermophyton* 3/29 (10.3%), *Microsporum* 9/29 (31.1%) and *Trichophyton* 17/29 (58.6%). Among the age group of 11–15 years old patients, *Trichophyton* 6/9 (66.6%) were most frequent while two of the samples that yielded no growth (Table 3).

**Table 3:** Distribution of putatively identified dermatophytes by age &sex of children visiting dermatology clinics in Hawassa (n = 83).

Variables	Dermatophytes			
	*Epidermophyton* (%)	*Microsporum* (%)	*Trichophyton* (%)	No growth (%)
Age (years)				
1–5	3 (10.3)	9 (31.1)	17 (58.6)	0 (0.0)
6–10	4 (8.9)	12 (26.6)	29 (64.4)	0 (0.0)
11–15	0 (0.0)	1 (11.1)	6 (66.6)	2 (1.0)
Sex				
Female	4 (7.8)	15 (29.4)	31 (60.7)	1 (2.0)
Male	3 (9.4)	7 (21.8)	21 (65.6)	1 (3.1)

The majority of the dermatophytes occurred in female patients 51/83(61.4%). Of the 51 samples from the female patients, 50 (98.04%) yielded growth on SDA. *Trichophyton* were 31/51 (60.7%), *Microsporum* were 15/51 (29.4%), and *Epidermophyton* were 4/51(7.8%). On the other hand, *Trichophyton* in males were 21/32 (65.6%), *Microsporum* were 7/32 (21.8%) and *Epidermophyton* were 3/32 (9.3%) (Table 3).

### Associated factors for dermatophytosis

The relation of dermatophytosis with categorical perceived factors among the study patients was analyzed by the chi-square test. Accordingly, age group (χ^2^ = 16.85, df = 2, p = 0.00) and history of migration from rural to urban (χ^2^ = 6.05, df = 1, p = 0.01) and site of infection (χ^2^** = **9.14, df = ,p = 0.01) were the only two risk factors found to be associated with dermatophytosis (Table 1).

## Discussion

In our study 83 clinically suspected patients of cutaneous mycosis were included and skin, hair and nail samples were obtained during the study period. The direct KOH mount microscopic examination of clinical specimens showed that all of the study participants (83/83 or 100%) were positive for dermatophytosis. However, the culture of the specimens on SDA showed growth only for 81 (97.6%) of the patients. Due to a lack of supply for staining and facility for molecular further identification to species level was not conducted. In agreement with the present observation, a higher detection yield of dermatophytosis by the KOH mount and microscopy than culture on SDA was reported in a study done in India where all the specimens from 165 study subjects (100%) were KOH positive while only 67.1% of them were culture positive^9^. Likewise, a study reported that of 131 dermatological samples investigated from school children in the Harari region of Ethiopia, 123 (93.8%) were KOH positive while 100 (76%) were culture positive^15^. In contrast, a study done in Iraq revealed that of 100 dermatological specimens investigated, 84% were KOH-positive while 93% were culture-positive^15^. Unlike, the report from Iraq, in the present study, the KOH mount and microscopy yielded a higher detection level than that from culture on SDA. These slight variations among the different studies might be due to differences in the Geographic location, living standards, culture, sample size and collecting site. It is widely accepted that low socio-economic status associated with large families in crowded living conditions, and poor hygiene, are associated with increased prevalence of dermatophytosis^16^.

In the present study, Tinea capitis was the main clinical manifestation agreeing with a study on African children^17^. A study in Egypt reported Tinea capitis (85.2%) followed by Tinea corporis (8.1%) and *Tinea unguium* (6.7%) in order were the most frequent cases^18^. This is concordant with our study. Tinea capitis is an infection of scalp hair follicles and the surrounding skin caused by dermatophyte fungi, usually by *Microsporum* (ectothrix) and *Trichophyton* (endothrix) and its clinical presentation are highly variable, depending on the causative organism, type of hair invasion, degree of host inflammatory response^19^. It is a type of dermatophytosis presentation most commonly seen in children^20–22^ and the data in the present study is, therefore, in agreement with this general norm. Although dermatophyte infection of the general body surface, tinea corporis is also known to be fairly common in children, it constituted a minor observation in the present study. On the other hand, nail infection (onychomycosis or *Tinea unguium*) is unusual during the first two decades of life, with a prevalence of less than 1% that increases progressively with age^22^. The data in the present study is concordant with the above observation.

The etiological agents of tinea capitis are known to exhibit successional changes over time and geographical region. For example, in the 1960 and 1970s, the less severe and transmissible *Microsporum* species were the dominant species that caused infection of the scalp in North America. It is now replaced by the rising dominant species of *Trichophyton tonsurans*, which is the most common cause of tinea capitis in the region. *T. tonsurans* has also been reported to be on the rise in urban areas of London, UK^23^, and Parisian areas in France^23^. In Africa, other *Trichophyton* species like *T. Verrucosum* and *T. violaceum* are the more common causes of tinea capitis than *T. tonsurans*^20^. A study done in Uganda based on 115 patients aged 1–16 years, reported *T. violaceum* (56.6%) as the most common cause of tinea capitis followed by *Microsporum audouinii* (13%)^24^. Unlike the general geographic distribution of the species reported before, *Trichophyton* genera were the predominant species of dermatophyte isolated from hair/scalp samples in the present study. Being strictly general is subject to wide distribution following human migration. It has been observed that *T. rubrum* and *T. tonsurans*, are now cosmopolitan but appear to have had a more restricted distribution in the past, having been transported widely as a result of human migration^21^.

In agreement with the present study, a study from Kenya reported *T. tonsurans* under the genera of *Trichophyton* was the predominant cause of tinea capitis^25^. *Trichophyton* has never been reported as a predominant cause of tinea capitis in children in Ethiopia before. In a similar study done in Tikur Anbessa Teaching Hospital, Addis Ababa, Ethiopia based on samples from 305 children investigated, *T. violaceum*, *T. mentagrophytes* and* M. canis* in order were the reported dominant dermatophytes that caused tinea capitis^9^. Another study done in Harari Regional State of Ethiopia reported *T. rubrum*, *M. audouinii*, *T. violaceum T. mentagrophytes*, *E. floccosum*, and *M. canis* in order were the predominant causes of tinea capitis in children^26^.

In contrast with the present study, the majority of the studies done in Africa reported a preponderance of dermatophytosis in male than female children^17^. A few studies done in Ethiopia also reported dermatophytosis is more in male than female children. A study in the Harari region of Ethiopia reported that of the 100 dermatophytosis culture-positive school children, the majority were males (52%) and 48% females^26^. *Tinea capitis* was the predominant presentation and *T. violaceum* and *T. rubrum* in order were the most frequent isolates from both sexes. In another study done in Ethiopia, Woldeamanuel et al. also reported dermatophytosis more in male than female children^27^. One study done among 428 school children in Harari Regional State of Ethiopia, reported that 100 (23.4%) of them had culture-confirmed dermatophytosis of which *tinea capitis* accounted for 77 of them the major causative agents were *T. violaceum* (43/77 or 55.84%) and *T. rubrum* (24/77 or 31.17%) under genera of Trichophyton. The majority of culture-confirmed cases were in the age group of 10–14 years (62%) while 38% were in the age group of 5–9 years old. The present study is slightly in contrast with the report from the Harari region in that younger age groups (less than 10 years old) were the most affected^26^. A study done in Nairobi Kenya reported the isolation of dermatophytes from 150 samples and the most frequent cases were among 9–11 years patients followed by the age group of 6–8 years and ages groups of 12–14 and 3–5 years. Precise comparison is difficult since age grouping differed among the studies. But again this report from Kenya may be considered concordant with the present observation in that patients younger than 10 years old were the most affected^19^. A similar study in Nigeria based on an investigation of 100 samples reported that dermatophytosis was most frequent among the age group of 11–15 years (50%) old followed by that of 5–10 years (42.6%) old study patients^28^. Another study from Nigeria also reported that the majority of the children with dermatophytosis in their study were in the age group below 10 years 41(83.7%)^29^.

In agreement with the present study, the predominance of dermatophytosis is more in females than males among school children in reported in Alexandria, Egypt^30^. Likewise, a study done in Brazil among 590 children in the age group of 12 years and under, investigated dermatophytosis by the culture of the different specimens. The study revealed that 210 of the samples were positive consisting of 125 (59.52%) females and 85 (40.48%) males. The majority of the cases were *tinea capitis* (153) followed by *Tinea corporis* (48) and *Tinea pedis* (6). The principal causative agents of *Tinea capitis* were *T. tonsurans* (121/153 or 79.1%) and *M. canis* (24/153 or 15.69%)^31^. The observed variation in the incidence of dermatophytosis by sex among the different studies might be due to differences in culture, and religion, among the study populations.

There was a statistical association between dermatophytosis incidence and the age of children, site of infection and migration from rural to urban (p < 0.05). A similar study done in Harari Regional State of Ethiopia reported observation in agreement with this study^26^. A similar study from the Netherlands also reported an association of dermatophytosis with rural-to-urban migration^32^. Likewise, a study in California reported the most important predictor of *Tinea capitis* is migration and the most likely site of infection was hair/ scalp in children^28^.

## Limitations of the study

In the present study confirmation of species by observation of micro- and macroconidia was not possible due to a lack of reagents and facilities like lactophenol cotton blue stain and molecular identification tools. Besides this, this study can’t confer the whole situation in the area as it is a cross-sectional study.

## Conclusion

The KOH mount and microscopic examination of dermatological specimens yielded a higher number of cases than the culture method in the present study. However, the culture method has the added advantage of observation of colonial morphology and putative identification of the etiological agent with the help of a standard colour atlas. Therefore, the use of both methods is recommended in the diagnosis of dermatophytosis and further study with a large population with advanced testing is recommended in future by using these findings as a baseline to determine the burden of dermatophytes in different populations.

## Data Availability

The datasets used and/or analyzed during the current study are available from the corresponding and main author upon reasonable request.
